# Supplementary figures and images for: Comparison of sequence- and structure-based antibody clustering approaches on simulated repertoire sequencing data
Source: PLoS Comput Biol. 2025 May 30;21(5):e1013057. doi: 10.1371/journal.pcbi.1013057 (PMC12148228; doi:10.1371/journal.pcbi.1013057)

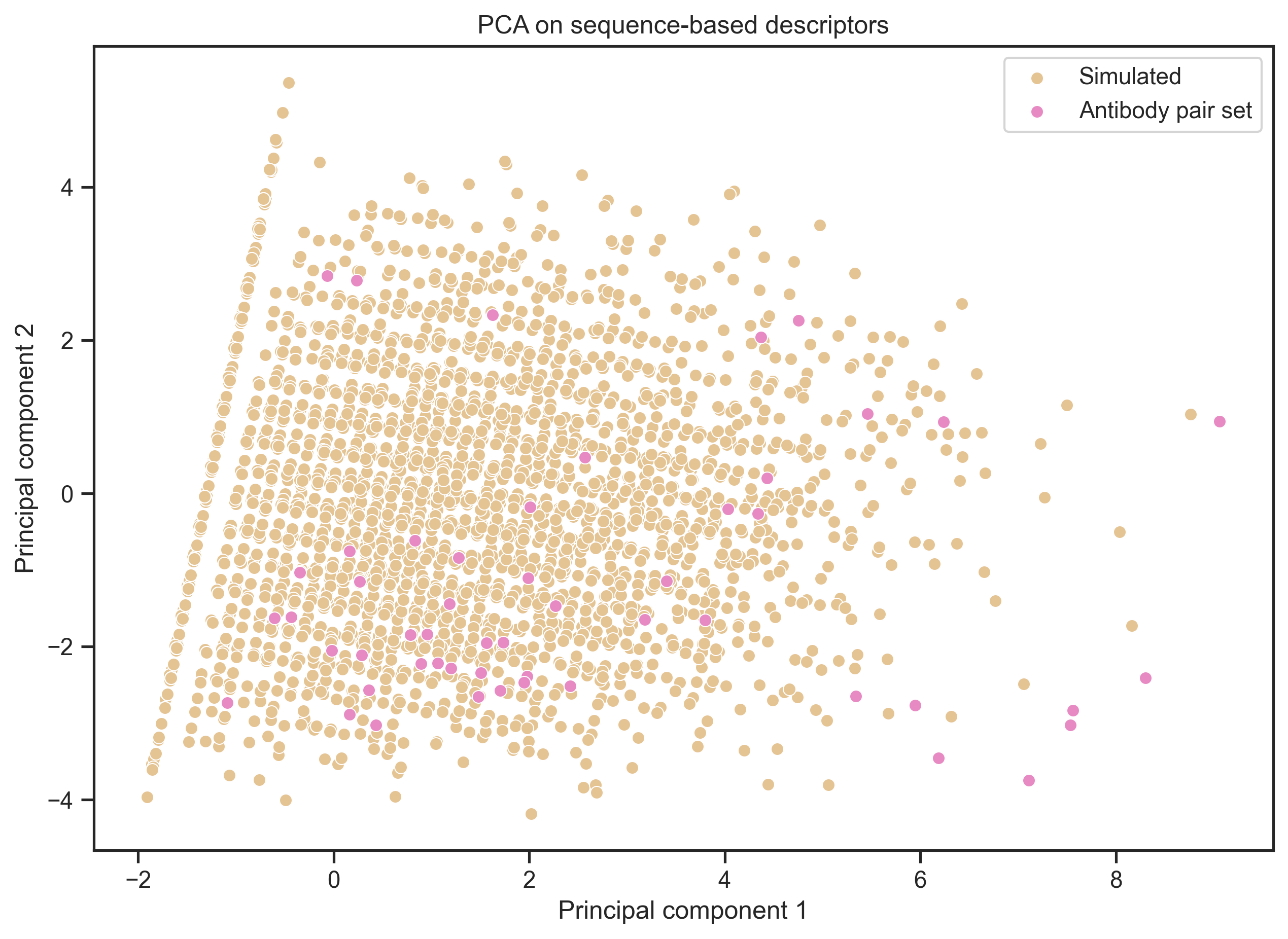

Supplement: S1 Fig — A PCA was fitted using sequence descriptors of the simulated repertoire and the antibody pair set. The first and second principal component are shown. The annotated (pink) and simulated (beige) antibodies overlap strongly, indicating that these antibody sets are not consistently different from each other. (TIF) [file pcbi.1013057.s001.tif]

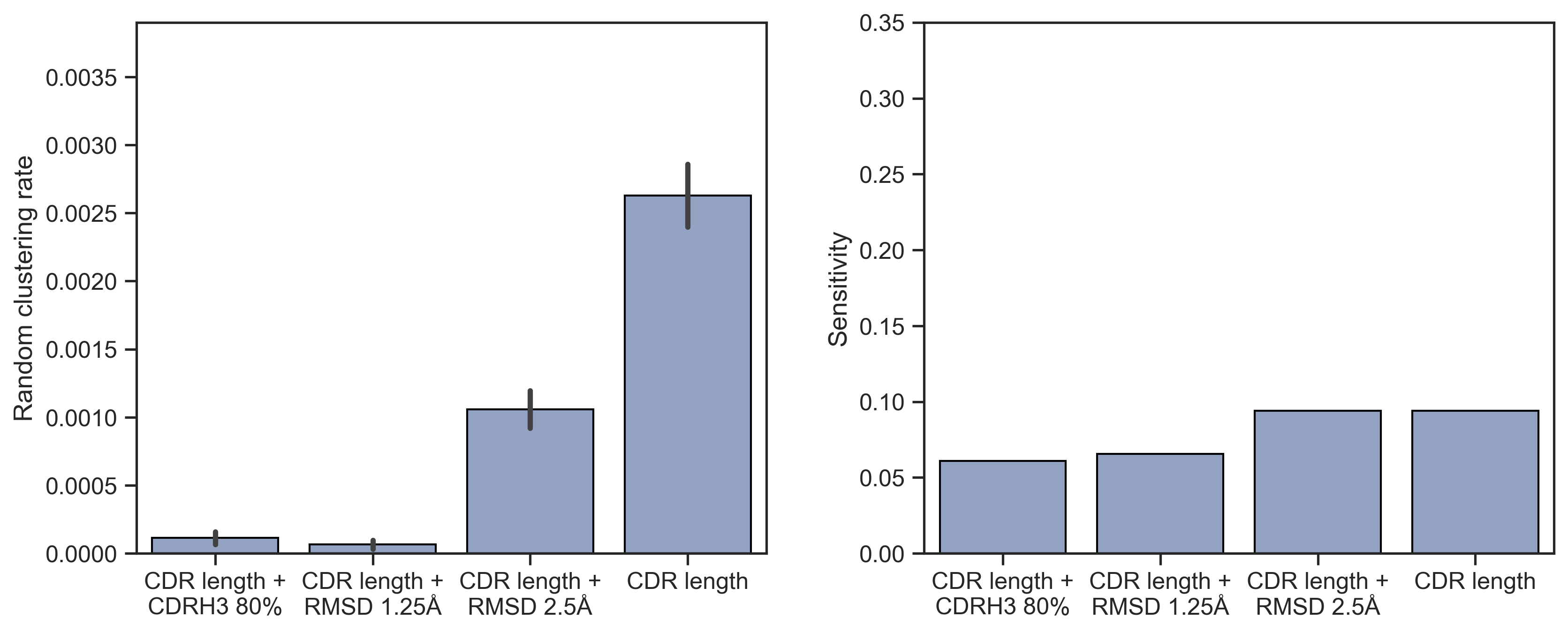

Supplement: S2 Fig — A: The random clustering rate is significantly higher when clustering is solely based on CDR length, compared to approaches that incorporate structural or CDRH3 sequence information as well. B: Combination of CDR length and CDRH3 sequence identity leads to the lowest sensitivity. Relaxing the RMSD cutoff increases sensitivity until all identical CDR lengths antibody pairs are correctly identified. (TIF) [file pcbi.1013057.s002.tif]
