# Supplementary material for: Comparison of sequence- and structure-based antibody clustering approaches on simulated repertoire sequencing data
Source: PLoS Comput Biol. 2025 May 30;21(5):e1013057. doi: 10.1371/journal.pcbi.1013057 (PMC12148228; doi:10.1371/journal.pcbi.1013057)
Supplement: Attachment — Submitted filename: Response_to_reviewers_PLOSCB.pdf [file pcbi.1013057.s005.pdf]

Response to reviewers

Reviewer #1

In this paper Waury et al. investigate the utility of antibody structural clustering methods, specifically SAAB+ and SPACE2, for their ability to group sequences that bind to the same epitope on a given antigen amidst a diverse simulated repertoire. They compare these two structural clustering methods to the traditional method of 'clonotyping' which relies on the CDRH3 sequence identity and V/J genes to group similar sequences. Structural methods instead group by shape of the paratope region and therefore can identify relationships which may not be evident from sequence alone. SAAB+ relies on homology modelling whereas SPACE2 clusters length matched CDRs. The ability of these methods to group pairs of antibodies which bind the same epitope amidst a large artificial repertoire was analysed.

No method was able to correctly group most of epitope-specific pairs antibody pairs. While clonotyping outperformed both structural methods, the authors demonstrated that structural clustering did group some pairs with highly dissimilar CDRH3 sequences. The limitations of each method are also assessed in detail. Of particular interest were observations the CDR length matching applied by SPACE2 is very restrictive, as well as the ability to increase cluster size (through lower stringency) without overly compromising specificity.

Overall, this paper is well thought out, figures are nicely presented, and the text is clearly written. I have only minor comments.

We are very happy to hear that our study was so well received and appreciate all the nice feedback as well as the helpful comments. We are grateful for the time spent reviewing this manuscript.

Line 199-201: Although the authors have tried to explain how pairs can be found in the clonotyping method that exceed the identity threshold, it is still not fully clear (assume they mean that within a cluster the overall distance might be greater, but distance from the cluster centroid is still below the threshold). Please spell this out exactly.

We agree that our explanation for clustered antibody pairs below the identity threshold was not clear enough in the original manuscript. We are hopeful that this revised, more illustrative paragraph is clearer:

"These antibody pairs of lower sequence identity are part of the large cluster of functionally related SARS-CoV-2 antibodies (Fig 1A). Within this cluster several antibodies form multiple antibody pairs allowing antibody pairs below the sequence identity threshold to be clustered together. To illustrate, antibody pairs A+B and A+C have a sequence similarity of 0.9 and are correctly clustered together by clonotyping. In this case, even if the antibody pair B+C has a low sequence similarity, e.g., of 0.7, this functional pair is still correctly clustered together because of their respective pairing with antibody A."

Line 435-437: In generating the artificial dataset. Does it matter that the authors have not used lambda chains and only focused on kappa? Please explain why?

The reviewer brings up an important point that we have not discussed previously in our manuscript. We have added the following paragraph to the discussion to bring attention to this limitation of our study:

“However, limitations of the simulated repertoire should be recognized. [...] Further, only kappa light chains were generated despite approximately 35% of human repertoires being comprised of lambda light chains [35]. This choice was made for simplicity considering that function is strongly driven by the antibody’s heavy chain [7]. However, the research bias regarding lambda light chains has been highlighted [35], underscoring the potential value of incorporating them in future work.”

Figure 2: Please state the statistical test used in the legend.

We have added the statistical test which was used to the figure description:

“Statistical significance was tested using the Wilcoxon rank-sum test.”

Line 116: Typo – should say clusters (plural).

We thank the reviewer for recognizing this typo and have corrected it.

Reviewer #2

The authors curated a dataset of antibody pairs confirmed to bind to the same epitope; they then placed these into a simulated naïve repertoire, clustered the simulated repertoire via 3 algorithms and looked at the sensitivity and specificity of these methods in the context of a naïve human background. In contrast to previous findings, they found that the sequence-based method had the highest sensitivity, and was able to identify a larger number of sequence-dissimilar/same-epitope pairs. The methodology is more precise and realistic than previous benchmarks, the claims are well-substantiated, and the dataset could be widely-used.

We are grateful that the reviewer appreciates this study’s methodology and the provided dataset and has suggested ways to improve our manuscript. We would like to thank the reviewer for the time and effort spent assessing our work.

Line 26 – DOI in text

We apologize for this oversight and have inserted the correct reference in the text.

Line 93 - Capitalize Immune Epitope Database

We thank the reviewer for noticing this error and have capitalized the database name.

Please cite the original SPACE paper.

We agree that the publication presenting the original SPACE method should be included in this work and have added it to the introduction at the end of the following sentence:

“As structure is more closely linked to function than sequence in folded proteins [13], using structural antibody information has received increased interest for the analysis of repertoire sequencing data [14–16].”

Line 58-59 - SPACE2 can work with any numbered antibody structure (predicted with ImmuneBuilder or otherwise), so this is not entirely accurate (although indeed this is recommended).

We thank the reviewer for this clarification and have updated the relevant section in the manuscript accordingly:

"SPACE2 requires numbered antibody structures as input such as those generated by the ab initio antibody structure predictor ImmuneBuilder [20, 24]."

Line 197 – Is this not just a consequence of hierarchical clustering?

We thank the reviewer for asking this clarifying question. We have revised the section explaining the low sequence identity antibody pairs clustered by clonotyping and hope the revised version is easy to follow and comprehend:

"Interestingly, clonotyping clustered antibody pairs below the threshold as well <u>because of its hierarchical clustering approach</u>. In fact, clonotyping identified more low-sequence similarity antibody pairs than SAAB+ and SPACE2 from our dataset (Table 2). <u>These antibody pairs of lower sequence identity are part of the large cluster of functionally related SARS-CoV-2 antibodies (Fig.~\ref{fig1}A). Within this cluster several antibodies form multiple antibody pairs allowing antibody pairs below the sequence identity threshold to be clustered together. To illustrate, antibody pairs A+B and A+C have a sequence similarity of 0.9 and are correctly clustered together by clonotyping. In this case, even if the antibody pair B+C has a low sequence similarity, e.g., of 0.7, this functional pair is still correctly clustered together because of their respective pairing with antibody A.</u>"

Figure 4 caption mentions specificity but I can't see this plotted anywhere (presumably because it is perfect at all thresholds)? It says "specificity stays high" – I would just say "specificity remains 100%".

We apologize for this confusion and the reviewer is indeed correct that this metric was not plotted as the value stayed at 100% across all tested settings. Based on the feedback of another reviewer we also decided to use precision (or positive predictive value) to discuss the prevalence of false positives within this analysis. Thus, the revised figure description contains the following sentence:

"Comparing different clustering settings shows that sensitivity can be increased while the precision remains at 100%, i.e., the number of falsely grouped antibody pairs does not increase."

I disagree with the usage of "functional clustering" to refer to non-validated clusterings in the simulated data - there is no evidence of shared function in these large clusters.

We thank the reviewer for bringing up this point. We have clarified this issue of "non-validated clusterings" within the discussion and hope the reviewer finds this sufficient:

"Namely, SAAB+ and SPACE2 grouped the antibodies into clusters of larger size. This could suggest that more functional convergence was detected compared to conventional clustering by clonotyping.

However, as annotations for the simulated repertoire are missing, no definite evidence of functional convergence is available."

It is not clear to me whether having any RMSD cut-off at all improves specificity, or whether most of SPACE2's predictive power is in CDR length matching. At the highest cut-off there is still perfect specificity. Please could you report sensitivity and specificity for the CDR length matching alone. Further to this - I haven't seen a simple CDR sequence identity clustering be compared to SPACE2. Please could you calculate the sensitivity+specificity using sequence identity instead of RMSD in the SPACE2 clustering algorithm- a threshold could be selected to produce a similar number of clusters to RMSD clustering or you could consider a reasonable range such as 70% and above. This would be stronger evidence that SPACE2 works because of structure specifically.

We are grateful to the reviewer for asking this important question. We have calculated the performance of two further approaches to cluster the repertoire antibodies as suggested by the reviewer: only using CDR length matching and using CDR length matching and a CDRH3 sequence identity of 80% or higher. The results have been included in Supplementary Fig 2 which compares the random clustering rate and the sensitivity with the structure-based approach of using CDR length matching combined with an RMSD cutoff of 1.25Å (SPACE2 default) and 2.5Å (clusters all possible antibody pairs together):

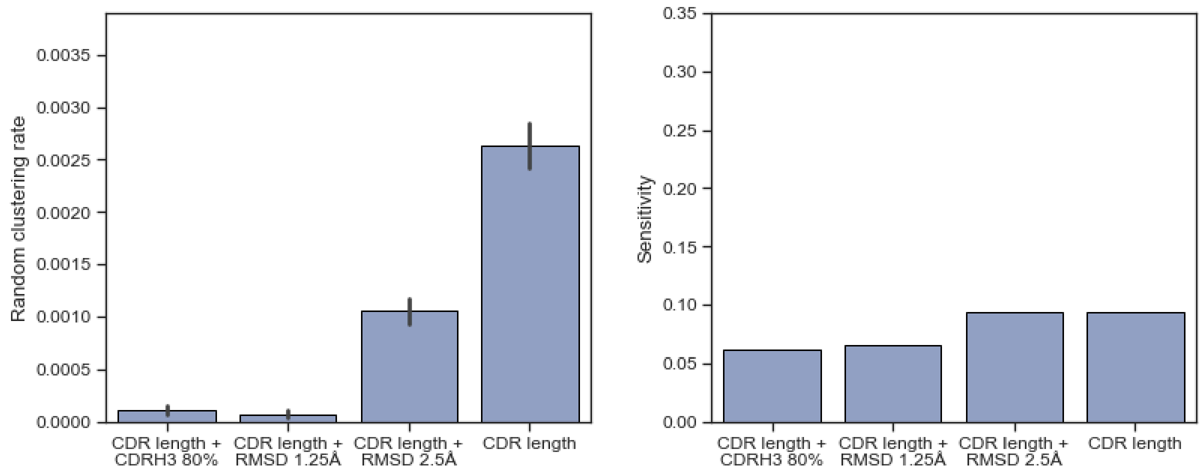

We further updated the manuscript's results and discussion to include this new analysis:

"To establish whether SPACE2 clustering works specifically due to the structural information, we compared the SPACE2 algorithm to an approach only utilizing the CDR length criterion and the CDR length criterion in combination with a sequence identity cutoff of 80% in the CDRH3 region. Neglecting structural information leads to a higher random clustering rate with identical sensitivity (S2 Fig), suggesting that the structure-based clustering does improve results. Further, clustering based on CDR length matching and CDRH3 sequence identity performs slightly worse than the default SPACE2 algorithm, both regarding the random clustering rate and the sensitivity (S2 Fig). None of these approaches cluster wrong antibody pairs."

"Additionally, we showed that combining the CDR length matching with structural information was more efficient than adding sequence-based information (S2 Fig), suggesting that antibody sequence and structure contain complementary insights."

Reviewer #3

The manuscript by Waury et al. compares two recent approaches to detect functionally related antibodies from B cell repertoire data, SAAB+ and SPACE2 (refs. 22 and 23). The benchmark highlights several interesting features of these methods and points out significant limitations in applying structure-based algorithms to real repertoires.

A comparison of existing methods for detecting convergent evolution is important to researchers analyzing immune repertoires. However, the benchmark presented in this study is limited in scope as it only considers a single synthetic repertoire with a trivial clonal structure. Some of the conclusions, in particular regarding the comparison of structure-based methods with clonotyping, are poorly supported by evidence presented in the manuscript.

I think two factors significantly diminish the methodological insight of this paper and its potential impact.

We are grateful that the reviewer acknowledges the importance of this method comparison for immune repertoire analysis. We appreciate the comments which allowed us to improve defining the scope of our work and highlight the limitations. We are convinced that the points raised are important to consider and should be the focus of future research. We thank the reviewer for the thoughtful comments and their time.

A single synthetic dataset was used for comparison and its parameters don't reflect the potential difficulties in detecting convergent evolution. These include varying degrees of mutation, repertoire size (depth of sampling), and, seemingly most important for the task at hand, a nontrivial clonotype structure. Antibody repertoires come in multiple shapes and sizes, and a meaningful comparison of clustering approaches must take that into account.

We appreciate that the reviewer brings up this important point and we agree that one dataset may not fully cover the wide range of possible characteristics of B-cell repertoires. However, we did spend effort creating a realistic repertoire dataset which (as we mentioned in the Introduction), and is - to our knowledge - the most complex dataset used in a study evaluating structure-based tools. Our dataset contains functionally related antibodies that were either produced by somatic hypermutations or functional convergence by including high and low sequence similarity antibody pairs. Further, we did spend effort on creating a dataset that does contain subsets of differing mutation rates. While these details were already explained in the methods section, we now realised that the manuscript might benefit from a short explanation in the results as well, which we have added in the revised version:

"The somatic hypermutation (SHM) rate of the simulated repertoire was set to match the SHM rates observed in the antibody pair dataset. In brief, a low and a high SHM rate was inferred from the antibody pair sequences; three repertoires of no, low and high SHM rate were then simulated and

combined. More details on the processes of sequence backtranslation and SHM rate calculation can be found in Methods."

Further, We have expanded our manuscript to discuss the limitations brought up by the reviewer and we hope they are satisfied with these additions:

"However, limitations of the simulated repertoire should be recognized. First, a single simulated repertoire cannot fully capture the diverse characteristics of B-cell repertoires. Although our dataset contains sequences with varying degrees of mutation rates, it does not account for repertoires of differing sizes or a non-naive background for the curated antibody pairs. Future studies applying these novel tools on additional repertoires, both simulated and native, can enhance our understanding of their capabilities and limitations."

The comparison with clonotyping is done with a relatively old method, which suffers from low accuracy, as suggested by more recent studies (see the following references and comparisons to other methods there: Ralph and Matsen, 2016 and 2022; Nouri and Kleinstein, 2020, Lindenbaum et al., 2020; Spisak et al., 2024), as well as by the results presented here (IGX-Cluster merges sequences that are not clonally related and have >40% divergence in CDRH3, in larger datasets this leads to positive predictive value close to zero).

We thank the reviewer for their comments regarding the appropriateness of our applied clonotyping approach. We would like to clarify that IGX-Cluster does indeed only cluster antibodies of the same genetic origin, as we required the V and J gene to be identical for two antibodies to be in the same cluster when comparing the three methods to one another. We apologize if there is any confusion about this clustering approach. Fig 3A highlights the shared V/J gene requirement as only antibody pairs from this group were clustered by IGX-Cluster. The misunderstanding regarding ">40% divergence in CDRH3" might stem from our explanation for low-sequence antibody pairs that were correctly clustered by IGX-Cluster. We have revised this part of the manuscript to clarify this point:

"These antibody pairs of lower sequence identity are part of the large cluster of functionally related SARS-CoV-2 antibodies (Fig.1A). Within this cluster several antibodies form multiple antibody pairs allowing antibody pairs below the sequence threshold to be clustered together. To illustrate, antibody pairs A+B and A+C have a sequence similarity of 0.9 and are correctly clustered together by clonotyping. In this case, even if the antibody pair B+C has a low sequence similarity, e.g., of 0.7, this functional pair is still correctly clustered together because of their respective pairing with antibody A."

In regard to the other clonotyping approaches that were suggested, we are thankful to the reviewer to share these references with us and we have reviewed these papers. The field of clonal family inference algorithms is an active research field as these recent publications showcase. However, we do not deem these alternative approaches to be highly relevant to this work. In this study we aimed for a comparison of novel structure-based approaches to an established sequence-based baseline approach. A comprehensive comparison and benchmark of various clonotyping methods is outside the scope of this work and has been attempted by other researchers recently (Balashova et al., 2024, DOI: 10.1186/s12865-024-00600-8). While advances in detecting clonal families are important for BCR repertoire analysis, even more

accurate methods to identify clonally related families still suffer from the limitations of not detecting functionally converged antibodies. Thus, we do not think that the use of IGX-Cluster is a stark limitation to the methodology as the focus is not on optimizing the identification of clonal families. Rather, we wished to evaluate the methods' identification of functionally related antibodies. We have revised the Introduction to make the aim of this study clearer:

"Thus, the repertoire may contain antibodies that have "functionally converged" despite their genetically differing origins [8]. Identification of antibodies with the same binding specificity, also called "paratyping", is of high interest to discover and engineer novel therapeutic antibody candidates [12]. Clonotyping would inadvertently overlook some of them because of the reliance on sequence similarity to define groups."

"Most importantly, it is vital to confirm if these novel types of approaches are able to identify functionally converged antibodies while also grouping clonally-related antibodies."

"The further focus of analysis revolves around the capabilities of the different approaches to accurately identify and group functionally related antibody pairs together, both clonally related and evolutionary converged ones. Thereby, this study provides insights into their effectiveness in capturing relevant structural and functional relationships within a diverse antibody repertoire."

We have added a paragraph to the Discussion regarding alternative approaches to identify clonal families to provide the interested reader with a wider view of the field and its current advances:

"In this case, clonotyping will provide the most suitable grouping and will allow further downstream analysis providing insights into the immune response, e.g., clonal expansion events. Note that the accurate identification of clonal families is an active research field with many approaches having been suggested in recent years that maximize accurate inference of antibodies with the same genetic origin [41, 42]."

The paper is appropriately structured. Data and code are readily available and the methodology is clearly described in the manuscript. The clarity of the presentation could be improved by avoiding the use of jargon. A few concrete suggestions and comments are listed below.

We are happy to hear that the reviewer approves of many parts of the manuscript and are grateful for these positive comments. We apologize that the clarity of the text suffered by the use of jargon. We have read through the entire manuscript again to find and improve potential issues and have kindly used the feedback provided below to improve readability.

Abstract: I'm afraid the phrase "multiple-occupancy clusters" will be confusing to the reader, and probably shouldn't be introduced in the abstract. I find the formulation in "author's summary" easier to follow.

We thank the reviewer for their feedback and have updated the relevant part of the abstract accordingly:

"Our analysis reveals that structure-based methods do group more antibodies together compared to clonotyping."

Note, however, that we are still introducing the term "multiple-occupancy clusters" later within the manuscript as this term was used in relevant previous publications, most notably the paper describing SPACE2 (Spoendlin et al., 2023).

The reference in line 26 is not formatted.

We apologize for this oversight and have inserted the correct reference in the text.

In line 27: "them" suggests "some of them".

We thank the reviewer for this suggestion and have updated the sentence:

"While identification of antibodies with the same binding specificity (sometimes called "paratyping") is of high interest, clonotyping would inadvertently overlook some of them because of the reliance on sequence similarity to define groups."

In line 84: this sentence is hard to parse

We agree that his sentence is not easy to read in the original version. We hope the revised sentences are easier to parse:

"This approach allows to assess the performance on a diverse and non-enriched set of BCR sequences. In addition, a baseline for "random" clustering rates can be established across all included methods."

In line 95: the 75% threshold is not justified.

We agree with the reviewer that a justification for the threshold chosen for the epitope overlap of included antibody pairs is missing. We have added this explanation within the results section:

"If the overlap of the residues of two epitopes was ≥75% as defined by their Jaccard index, the respective antibodies were defined as functionally similar and retained. This overlap cutoff allowed the inclusion of a sufficient number of antibody pairs."

"This observation indicates that our chosen epitope overlap cutoff of ≥75% ensures that all included antibody pairs are functionally similar enough to be grouped together."

When discussing sequence identity, it should be stated whether this refers to amino acid or nucleotide sequence.

We have added the information that the sequence identity is calculated at amino acid level to the introduction:

"Commonly, a CDRH3 sequence identity of ≥80% at the amino acid level is used to group antibodies as one clonotype together, although a higher cutoff can be applied if warranted [5]."

We have also included the following statement in the methods section:

"Unless stated otherwise, sequence identity is calculated at the amino acid level."

In line 133: suggest avoiding "spiked"

We thank the reviewer for this suggestion and have replaced the term "spiked" with "introduced":

"The reconstructed antibody pair nucleotide sequences were then introduced into the simulated repertoire resulting in our full repertoire dataset of 10.490 unique sequences."

In line 149: The sentence "Antibodies (...)" is not grammatical.

We apologize for the sentence not being correct and have included the following in the revised manuscript:

"Antibodies were annotated from the sequencing data, with the closest V and J reference gene, as well as somatic hypermutations being identified."

In line 167. One cannot infer that any of the methods is highly specific from the observation that no two curated antibodies were incorrectly assigned to the same cluster. More generally, in discussing the performance of the methods, it's not specificity but positive predictive value or precision, that's 1. a relevant measure of accuracy and 2. difficult to achieve in clonotyping.

We are grateful to the reviewer for bringing up this point and agree that precision (or PPV) is more suitable to use within our manuscript to discuss the prevalence of false positive antibody pairs. We have exchanged the correct term throughout the manuscript to discuss precision instead of specificity where appropriate. For example:

"On the other hand, all three methods show high precision as no two antibodies were incorrectly assigned to the same cluster."

In line 184: suggests rather "identified by"?

We thank the reviewer for this suggestion and have updated the sentence accordingly:

"Comparing the epitope overlap of the antibody pairs identified by each method shows no strong difference between methods and the full antibody pair dataset (Fig 2D)."
